# Assessing provider and racial/ethnic variation in response to the FDA antidepressant box warning

**DOI:** 10.1111/1475-6773.13104

**Published:** 2019-01-21

**Authors:** Benjamin L. Cook, Ye Wang, Rajan Sonik, Susan Busch, Nicholas Carson, Ana M. Progovac, Alan M. Zaslavsky

**Affiliations:** ^1^ Health Equity Research Lab Cambridge Health Alliance Harvard Medical School Cambridge Massachusetts; ^2^ Disparities Research Unit Massachusetts General Hospital Boston Massachusetts; ^3^ Tucker‐Seeley Research Lab Leonard Davis School of Gerontology University of Southern California Los Angeles California; ^4^ Yale School of Public Health New Haven Connecticut; ^5^ Department of Health Care Policy Harvard Medical School Boston Massachusetts

**Keywords:** racial/ethnic differences in health and health care, modeling: multi‐level, psychiatry

## Abstract

**Introduction:**

After the 2004 FDA box warning raised concerns about increased suicidal ideation among youth taking antidepressants, antidepressant use decreased for White youth but slightly increased for Black and Latino youth. Better understanding of patient and provider factors contributing to these differences is needed to improve future risk warning dissemination.

**Methods:**

We analyzed antidepressant prescriptions for youth aged 5‐17 in 2002‐2006 Medicaid claims data from four states (CA, FL, NC, and NY). In multilevel models, we assessed provider‐ and patient‐level contributions to changes in antidepressant use by race/ethnicity and compared responses to the box warning between providers with large (>2/3) and small (<1/3) proportions of minority patients.

**Results:**

A significant amount of variance in overall prescribing patterns (calculated by the ICC) was explained at the provider level. Significant provider‐level variation was also identified in the differential effect of the box warning by racial/ethnic group. In a test of the influence of provider panel mix, we found that providers with large proportions of minority patients reduced antidepressant prescribing more slowly after the box warning than other providers.

**Discussion:**

This study is the first to assess provider‐ and patient‐level variation in the impact of a health care policy change on treatment disparities. Black and Latino youth Medicaid beneficiaries were seen by largely different providers than their White counterparts, and these distinct providers were influential in driving antidepressant prescription patterns following the box warning. Concerted outreach to providers of minority beneficiaries is needed to ensure that risk warnings and clinical innovations diffuse swiftly across racial/ethnic minority groups.

## 1 INTRODUCTION

The Food and Drug Administration (FDA) utilizes box warnings to disseminate information regarding risk of adverse effects associated with medication use. The issuance of box warnings has led to significant declines in overall use of the targeted drugs;^1^, ^2^, ^3^ however, the diffusion of new safety guidelines takes time^4^ and occurs at differing speeds across racial/ethnic groups, which may result in a lesser quality of care for racial/ethnic minority patients.^5^, ^6^ After the 2004 box warning on antidepressants warned of an increased risk of suicidal ideation among young people, rates of antidepressant use significantly decreased for White youth, but slightly increased for Black and Latino youth,^7^, ^8^ leading to a reduction in the racial/ethnic *difference* 1 in antidepressant use. The 2004 antidepressant box warning provides a relevant case study to investigate how warnings about the risks of medication diffuse differentially across patients from different racial/ethnic groups, and may provide important lessons for understanding efforts to stem the inappropriate prescription of opiate pain medications and other medications (eg, benzodiazepines) with high risk of abuse, addiction, or negative side effects.

Whether a racial/ethnic minority youth with depression is prescribed an antidepressant is likely to depend largely on the provider from whom they receive care. In studies of adult patients, providers that treat predominantly racial/ethnic minority patients are less likely to be influenced by new and emerging external scientific evidence compared to providers that treat predominantly White patients.^9^, ^10^ Similarly, it has been found that clinicians in health care settings that serve diverse patients are less likely to employ evidence‐based practices (EBPs).^11^, ^12^, ^13^ As such, guidelines published by the FDA and other organizations may be less likely to influence treatment patterns of providers of racial/ethnic minority patients than providers of non‐Latino White patients.

Ideally, providers and patients (and caregivers of youth patients) make decisions jointly about the treatment plan.^14^ However, providers often use medical language that patients do not understand, and rarely ask patients about their preferences with respect to medications, therapies, and procedures.^15^ Provider‐patient communication is even worse among more marginalized patient groups such as racial/ethnic minorities.^15^ Clinicians may be less likely to adequately communicate potential risks associated with a particular medical treatment to minority patients than White patients due to cultural and linguistic barriers.

Providers with specialty training in mental health (ie, psychologists, psychiatrists, social workers) are more likely to effectively receive, interpret, and disseminate information regarding EBPs than are pediatricians and other primary care providers (PCPs). This suggests that youth that receive mental health treatment from only a PCP (approximately 1/3 of all youth receiving mental health treatment^16^) may be at a greater risk for missing key risk information. This may be of greater concern for racial/ethnic minority youth given that they are more likely than Whites to seek mental health care with PCPs than specialists.^17^, ^18^ Furthermore, racial/ethnic disparities in diagnosis and treatment (through counseling and/or medication) of anxiety and depression are greater in primary care than in psychiatric service settings.^19^ The reduced access to and utilization of specialty mental health care providers for racial/ethnic minority youth may thus present additional obstacles to effective dissemination of risk information to minority patients and their caregivers.

At the patient level, there are several reasons why racial/ethnic minority youth patients and their caregivers may be less likely than White youth patients and their caregivers to respond to an FDA risk warning. Firstly, racial/ethnic minorities tend to receive health information from different sources than Whites and are less likely to trust the information that they receive.^20^ While there was significant coverage of the box warning in news outlets and the popular press,^21^ racial/ethnic minority families may have been less exposed to this coverage. Secondly, many consumers avoided depression treatment altogether after the box warning.^22^, ^23^, ^24^ If avoidance of depression treatment occurred more frequently among Whites than Blacks and Latinos post–box warning, this could explain the greater decline in antidepressant use in White patients. Thirdly, racial/ethnic differences in the diffusion of risk warnings may be influenced by the caregiver's level of education,^25^ and the youth's medical history,^26^ severity of mental illness, and health insurance plan,^27^ all factors that typically differ by race/ethnicity.

The youth antidepressant box warning provides a case study to improve our understanding of how providers, patients, and their caregivers respond to risk warnings, and how the racial/ethnic makeup of a provider's patient panel influences treatment after a risk warning. Using multilevel models accounting for nesting of treatment events within patients within providers, we evaluated how the effects of the box warning differ between providers and the amount of variation that is explained at the provider and patient levels. Assessing the provider‐level random effects in these multilevel models, we test the hypothesis that significant variation in prescribing patterns exists *between* providers and *within* providers, and test whether additional provider‐level variation over and above race and time effects exists to explain the differential reactions to the antidepressant box warning. Then, to provide more detail on the provider‐level influence on the diffusion of this policy change, we test the hypothesis that providers that treated predominantly White patients were more likely to decrease rates of antidepressant prescriptions post–box warning compared to providers that treated predominantly minority patients.

## 2 METHODS

### 2.1 Data

Data are a 10 percent sample of the Medicaid Analytic eXtract (MAX) files for California, Florida, New York, and North Carolina for the years 2002‐2006. These four states were chosen because they are representative of other racially/ethnically diverse states, their large Medicaid beneficiary populations (these states make up over 33 percent of the entire Medicaid population of the United States), and their relatively low rates of Medicaid managed care penetration during the years of study, allowing for a focus on fee for service beneficiaries for whom MAX contains complete data. The MAX data are extracted annually from the Medicaid Statistical Information System (MSIS) and include information on beneficiary demographic information, services used, and medications supplied, as well as International Classification of Diseases, Ninth Revision (ICD‐9) diagnostic codes for each service provided. We linked services rendered and prescription drug claims to state provider identification numbers. The final dataset merges the outpatient and pharmacy MAX files and includes eligibility, encounter, and pharmacy data for all youth aged 5‐17 years enrolled in Medicaid programs in these four states during the study period. We defined three time periods: pre–box warning (January 2002 to September 2003), “phase‐in” (October 2003 to October 2004), and post–box warning (November 2004 to December 2006). In October 2004, the FDA issued the warning that antidepressants were associated with an increased risk of suicidal ideation in youth. We excluded claims from the “phase‐in” period because of preliminary FDA advisories starting in October 2003 that could have affected provider awareness of suicidality concerns and prescribing of antidepressants.

The sample consists of claims from non‐Latino White, Black, and Latino youth eligible for Medicaid in each month of analysis, totaling 7 026 029 claims for outpatient visits, inpatient visits, and medication fills, attributed to 1 228 596 Medicaid beneficiaries (365 209 White; 293 782 Black; and 569 605 Latino). We excluded Asian, Native American, and multiracial youth due to small sample sizes.

Claims were aggregated to the patient‐month unit of analysis; each observation contains antidepressant data for a given month for a specific patient. For each patient‐month, we assigned the patient to the provider that he/she saw for the greatest percentage of their outpatient visits in that calendar year. In the case of ties, one of the tied providers was randomly chosen. If there were no visits during the year or there was no available provider identifier, then the patient data were not used for that year. The list of antidepressants was determined using Multum medication classification codes.

This data structure allows for multilevel modeling, which can account for the nonindependence of multiple months of data from the same individual.^28^ Further, this modeling strategy allows for investigation of provider‐level differences that are not possible in the uni‐level difference‐in‐differences and interrupted time series models used in prior box warning studies. In particular, it allows testing of whether data from multiple individuals seeing the same provider are nonindependent. A further benefit of multilevel models is that they use data from all available individuals and providers, even those with missing data in some of the time periods.

### 2.2 Model

We implemented a multilevel linear probability model to examine the differential impact by race/ethnicity of the box warning (equations (1a), (1b), (1c), (1d), (1e)), estimating a similar model specification as prior studies but with careful attention to the nesting of multiple patient observations within patients within providers. The first main analysis of interest was to use the multilevel framework to assess the independence (or nonindependence) of observations from patients seen by the same provider. To do so, we added *u*_00*j*_ a provider‐level random intercept (equation 1f) and calculated an intraclass correlation coefficient to measure the variance between and within providers. The second analysis of interest was to assess whether the interaction between race/ethnicity and the change in the slope of antidepressant use post–box warning (β_31*j*_ in equation (1e)e) varied significantly between providers. This identifies the provider‐level variation in the differential effect of the box warning by racial/ethnic group. For this step, we used likelihood ratio (LR) tests to iteratively test the significance of provider‐level random effects *u*_01*j*_ through *u*_30*j*_ (in equations (1g), (1h), (1i), (1j), (1k), (1l)), ultimately building to a likelihood ratio test comparing models with and without *u*_31*j*_ (a provider‐level random effect on β_31*j*_) in equation 1m below.

Level 1 (time):

(1a) $$\begin{array}{cc} P(Y_{\mathrm{tij}} & =1)=\pi_{0ij}+\pi_{1ij}*{\text{Months}}_{\mathrm{tij}}+\\ & \pi_{2ij}*{\text{Post‐BW}}_{\mathrm{tij}}+\pi_{3ij}*{\text{Months‐Post‐BW}}_{\mathrm{tij}}+e_{\mathrm{tij}} \end{array}$$

Level 2 (patient):

(1b) $$\pi_{0ij}=\beta_{00j}+\beta_{01j}*{\text{race}}_{.ij}+r_{0ij}$$

(1c) $$\pi_{1ij}=\beta_{10j}+\beta_{11j}*{\text{race}}_{.ij}$$

(1d) $$\pi_{2ij}=\beta_{20j}+\beta_{21j}*{\text{race}}_{.ij}$$

(1e) $$\pi_{3ij}=\beta_{30j}+\beta_{31j}*{\text{race}}_{.ij}$$

Level 3 (provider):

(1f) $$\beta_{00j}=\gamma_{000}+u_{00j}$$

(1g) $$\beta_{01j}=\gamma_{010}+u_{01j}$$

(1h) $$\beta_{10j}=\gamma_{100}+u_{10j}$$

(1i) $$\beta_{11j}=\gamma_{110}+u_{11j}$$

(1j) $$\beta_{20j}=\gamma_{200}+u_{20j}$$

(1k) $$\beta_{21j}=\gamma_{210}+u_{21j}$$

(1l) $$\beta_{30j}=\gamma_{300}+u_{30j}$$

(1m) $$\beta_{31j}=\gamma_{310}+u_{31j}$$

*Y*_*tij*_ is the dichotomous dependent variable, coded as 1 if there is a filled prescription for an antidepressant for individual patient *i* from provider *j* at month *t*, and 0 otherwise. Months is a counter variable that equals 1 for the first month of the study period and increases by 1 for every month thereafter, Post‐BW is a 0/1 indicator of whether or not a given month was before or after the box warning came out, and Months‐Post‐BW is a counter variable that equals 1 for the first month after the box warning came out and increases by 1 for every month thereafter. Race/ethnicity (*race*) was determined using categories of non‐Latino White (“White”), non‐Latino Black or African American (“Black”), and Latino or Hispanic (“Latino”). The coefficients on the race_*ij*_ variables in level 2 represent interactions between race_*ij*_ and time variables. Covariances between random effects were assumed to be zero.

In the third analysis of interest, in order to better understand provider‐level variance, we tested the significance of observed provider‐level covariates, estimating a new multilevel model (equations (2a), (2b), (2c), (2d), (2e), (2f), (2g), (2h), (2i)). Specifically, we compared how the diffusion of the box warning across providers varied by the racial/ethnic makeup of their patient panels by estimating the following model, which simplified the patient‐level equations (1b), (1c), (1d), (1e) in order to focus on the provider level without excess interaction terms (equations (2f), (2g), (2h), (2i)):

Level 1 (time):

(2a) $$\begin{array}{cc} P(Y_{\mathrm{tij}} & =1)=\pi_{0ij}+\pi_{1ij}*{\text{Months}}_{\mathrm{tij}}\\ & +\pi_{2ij}*{\text{Post‐BW}}_{\mathrm{tij}}+\pi_{3ij}*{\text{Months‐Post‐BW}}_{\mathrm{tij}}+e_{\mathrm{tij}} \end{array}$$

Level 2 (patient):

(2b) $$\pi_{0ij}=\beta_{00j}+r_{0ij}$$

(2c) $$\pi_{1ij}=\beta_{10j}$$

(2d) $$\pi_{2ij}=\beta_{20j}$$

(2e) $$\pi_{3ij}=\beta_{30j}$$

Level 3 (provider):

(2f) $$\beta_{00j}=\gamma_{000}+\gamma_{001}*\left(\text{provider}\_{\text{comp}}_{..j}\right)+u_{00j}$$

(2g) $$\beta_{10j}=\gamma_{100}+\gamma_{101}*\left(\text{provider}\_{\text{comp}}_{..j}\right)$$

(2h) $$\beta_{20j}=\gamma_{200}+\gamma_{201}*\left(\text{provider}\_{\text{comp}}_{..j}\right)$$

(2i) $$\beta_{30j}=\gamma_{300}+\gamma_{301}*\left(\text{provider}\_{\text{comp}}_{..j}\right)$$

The categorical variable, provider_comp_..*j*_, represents the providers’ patient panel, consisting of >2/3, between 2/3 and 1/3, and <1/3 of racial minority patients (Black or Latino). The coefficients on the provider_comp_*..j*_ variables in level 3 represent interactions between provider_comp_*..j*_ and the time variables. In particular, we are interested in whether the reactions immediately after the box warning (*level shift*, γ_201_) and changes in the post–box warning slope (γ_301_) were greater or less among providers who had >2/3 racial/ethnic minority patients, as compared to providers who had <1/3 racial/ethnic minority patients. All models were estimated using the *STATA* 14 software package (StataCorp LLC, College Station, TX, USA).

## 3 RESULTS

We first describe, by race/ethnicity, the gender, Medicaid eligibility type, probability of any antidepressant fill, and age as of January 2002 or a patient's first appearance in the MAX data (Table 1). As has been found in previous studies, there is a decline in antidepressant use across racial/ethnic groups. We identified significantly smaller declines for Blacks compared to Whites. Latinos were more likely to be female and younger than Whites. Whites were significantly more likely to be eligible for Medicaid via Foster Care compared to Blacks and Latinos, whereas Blacks and Latinos were significantly more likely to be eligible via the Temporary Assistance for Needy Families program. Blacks were significantly more likely to be eligible via the Supplemental Security Income program than Whites, whereas Latinos were similar to Whites in this eligibility category.

**Table 1:** Socio‐demographic characteristics of a 10% random sample of NY, NC, CA, and FL Medicaid youth beneficiaries between 2002 and 2006 (n = 1 228 596)^a^

	Total	White	Black	Latino	*P*‐value for omnibus test of racial/ethnic differences
	(n = 1 228 596)	(n = 365 209)	(n = 293 782)	(n = 569 605)	
	%	%	%	%	
Rate of antidepressant use					
Pre‐BW	1.2	2.4	0.9	0.6	<0.001
Post‐BW	1.0	1.9	0.7	0.5	
Gender					
Male	49.9	50.2	50.1	49.5	<0.001
Female	50.1	49.8	49.9	50.5	
Age at first Medicaid enrollment					
5‐9 years	44.3	42.1	42.1	46.8	<0.001
10‐13 years	29.5	29.2	30.3	29.2	
14‐17 years	26.3	28.7	27.6	24.0	
Medicaid eligibility					
Foster care	4.9	8.2	6.0	2.3	<0.001
Temporary assistance for needy families	20.7	16.7	24.3	21.5	<0.001
Supplemental security income	5.0	4.9	8.6	3.2	<0.001
State children's health insurance plan	2.3	2.1	0.8	3.2	<0.001
Racial/ethnic composition of provider panel					
<1/3 Black or Latino youth patients	12.3	36.6	3.5	1.5	<0.001
Between 1/3 and 2/3 Black or Latino youth patients	22.4	34.6	26.0	12.7	
>2/3 Black or Latino youth patients	65.3	28.8	70.5	85.8	

a Summary statistics were calculated for unique Medicaid beneficiaries aging from 5 to 17 who had at least one outpatient claim observed in the MAX file and identifiable provider ID from 2002 to 2006.

A majority of all Medicaid youth beneficiaries (66 percennt) in this study, regardless of race/ethnicity, received treatment from providers that have panels composed of >2/3 Black or Latino patients (last row, Table 1). Parsed by race/ethnicity, 30 percent of Whites, 72 percent of Blacks, and 87 percent of Latinos received care from providers that have panels composed of >2/3 Black or Latino patients. In contrast, 36 percent of White beneficiaries, 3 percent of Black beneficiaries, and less than 2 percent of Latino beneficiaries visit providers that have panels with <1/3 Black or Latino patients.

In the first main analysis, we estimated a random intercepts model of antidepressant prescription to assess the overall intraclass correlation coefficient (ICC). The ICC indicates that provider‐level variation contributed 12 percent of the total variance in antidepressant prescribing compared to a contribution of 44 percent at the patient level, after conditioning on the race/ethnicity and time variables (Table 2). In the second main analysis, likelihood ratio tests for models that sequentially added provider‐level random effects (indicators of race, slope, and interactions between race and slope and change in slope) demonstrate that there is significant improvement in model fit as provider random effects on the Black race variable are added (Table 2; only Black interactions were reported for simplicity). The sequential likelihood ratio tests demonstrate that there continues to be significant improvement in model fit and thereby significant variation by provider underlying the differential box warning effect on Blacks over time.

**Table 2:** Intraclass correlation for provider random intercepts model and likelihood ratio tests assessing the significance of adding provider‐level random effects on each of the time and time*race/ethnicity covariates

Provider‐level random intercepts model, intraclass correlation, conditional on race, time, and race*time interactions		
Intraclass correlation at provider level	0.121	
Intraclass correlation at patient level	0.438	
Residual share in total variance	0.441	

Likelihood ratio tests	LR χ^2^	*P*‐value
Adding provider‐level random effect on Black race covariate	821.25	<0.0001
Adding provider‐level random effect on pre‐bw slope covariate	13 724.06	<0.0001
Adding provider‐level random effect on Black race*pre‐BW slope covariate	524.39	<0.0001
Adding provider‐level random effect on level shift covariate	3967.91	<0.0001
Adding provider‐level random effect on Black race*level shift covariate	29.25	<0.0001
Adding provider‐level random effect on change in slope covariate	9660.12	<0.0001
Adding provider‐level random effect on Black race*change in slope covariate	1774.05	<0.0001

Data: Medicaid analytic eXtract for NY, CA, NC, and FL (n = 7 026 029 medical claims for 1 228 596 beneficiaries).

Results in the third main analysis show that providers with >2/3 minority patients prescribed antidepressants at a lower rate than providers whose share of minority patients was less than 1/3 (Table 3). Consistent with our hypothesis, the interaction coefficients of the post–box warning slope change and “provider with >2/3 minority patients” were significantly positive, indicating that there were smaller rates of decline in antidepressant prescribing among providers with >2/3 minority patients after the box warning compared to providers with panels that had less than 1/3 minority patients.

**Table 3:** Multilevel linear probability regression assessing the influence of the 2004 antidepressant warning and provider panel composition on antidepressant prescriptions among youth Medicaid beneficiaries

	Coefficient	SE
Racial/ethnic composition of provider panel		
<1/3 Black or Latino youth patients	Referent	
Between 1/3 and 2/3 Black or Latino youth patients	−0.00410**	0.00049
>2/3 Black or Latino youth patients	−0.00914**	0.00040
Time main effects and time*provider panel interactions		
Pre‐BW Slope	0.00005**	0.00002
Interaction with panel between 1/3 and 2/3 Black or Latino youth patients	0.00004**	0.00002
Interaction with Panel >2/3 Black or Latino youth patients	−0.00001	0.00002
2004 Post‐BW Level Shift	−0.00113**	0.00047
Interaction with Panel Between 1/3 and 2/3 Black or Latino youth patients	−0.00165**	0.00060
Interaction with Panel >2/3 Black or Latino youth patients	0.00018	0.00051
Pre‐BW to Post‐BW Slope Change	−0.00010**	0.00002
Interaction with Panel Between 1/3 and 2/3 Black or Latino youth patients	0.000003	0.00003
Interaction with Panel >2/3 Black or Latino youth patients	0.00007**	0.00002
Constant	0.01181**	0.00036
Provider‐level random effects		
Intercept (SD)	0.00060**	0.00001
Patient‐level random effects		
Intercept (SD)	0.00217**	0.000003
Error residuals	0.00218**	0.000001

*Note:* The unit of observation is patient‐month.

Data: 2002‐2006 Medicaid analytic eXtract for NY, CA, NC, and FL (n = 7 026 029 medical claims for 1 228 596 beneficiaries).

**Significant at the *P* < 0.05 level.

## 4 DISCUSSION

This study is the first of which we are aware to assess provider‐ and patient‐level variation in the impact of a health care policy change on racial/ethnic treatment disparities. Future studies can consider use of these methods to identify levels or pathways by which other health care policies impact disparities in treatment. More specifically, we assessed the influence of provider‐level and individual‐level factors on racial/ethnic disparities in antidepressant use before and after the FDA box warning.

Similar to prior studies in the general population^7^ and among Medicaid beneficiaries,^8^ we found that overall antidepressant prescriptions declined significantly after the box warning among youth Medicaid beneficiaries in four states. Like prior studies, we identified a reduction in racial/ethnic differences in antidepressant use after the box warning because the decline in Whites was sharper than in Blacks and Latinos. This study builds upon these prior studies by using multilevel modeling, as opposed to uni‐level difference‐in‐difference and interrupted time series methods used in prior studies, allowing us to better understand the provider‐ and patient‐level contributions to the box warning impact on racial/ethnic disparities. This modeling strategy leads to three novel findings: (a) A significant amount of variance in prescribing patterns (as calculated by the ICC) was explained at the provider level, after conditioning on race, time, and race‐time interactions; (b) significant unexplained provider‐level variation related to the differential patterns in antidepressant usage by patient race/ethnicity both before and after the box warning (tested by sequential LR tests); and (c) antidepressant prescribing rates of providers with a large proportion of minority patients declined more slowly than the rates of providers with a smaller proportion of minority patients.

The unexplained provider‐level variation in our first model suggests that providers significantly affected the changes in antidepressant use after the box warning; however, several assumptions are required to equate provider variation with provider influence, including the critical assumption that physicians that had an effect on the shift were also responsible for changes in overall trends in antidepressant use. Alternatively, the strong influence by providers on the shift in antidepressant use could be due to the fact that patients of each provider were clustered among a small group of communities or neighborhoods with distinct attitudes concerning antidepressant use. In that scenario, the uniqueness of the composition of patients within providers’ panels, as opposed to the influence of the provider on those patients, explains our findings. We know of no prior studies describing significant regional or neighborhood differences among racial/ethnic minority patients in medication use, but we cannot rule out this possibility.

Racial/ethnic minority youth Medicaid beneficiaries receive treatment from a subgroup of providers that are different in terms of their panel composition than the subgroup of providers that treat White youth Medicaid beneficiaries, with 72 percent of Blacks, and 87 percent of Latinos receiving care from providers that have panels composed of >2/3 Black or Latino patients, compared to only 30 percent of Whites. This finding mirrors that of prior studies finding that differences in quality of care between racial/ethnic minority and White adult patients are to a large extent due to the fact that minority patients are treated by different groups of physicians^11^ and in different inpatient hospital settings.^29^ One implication is that policy makers concerned with equitable diffusion of innovation (or “exnovation”^30^ in the case of scaling back existing practices because of risk warnings) should consider interventions that intentionally reach out to providers that predominantly treat racial/ethnic minority patients. Furthermore, this provides evidence that de facto segregation across hospitals (due in part to differential physician referral practices and historical catchment areas that mirror residential segregation)^31^, ^32^ may extend to outpatient pediatric settings where psychotropic medications are prescribed.

The significant underlying provider‐level variation in prescribing patterns both before and after the box warning suggests that policies seeking equity in diffusion of innovation and risk warnings will need to target resources toward the outpatient facilities and community health centers that predominantly treat youth of color.

The fact that there also remains significant individual‐level variation in prescribing patterns suggests that there should be policies put into place that reduce potentially biased treatment patterns within these facilities and increase direct outreach and education to patients and their caregivers on the costs and benefits of new and high‐risk treatments. Future studies may benefit from exploring variation by provider type (eg, primary care physicians, psychiatrists, nurse practitioners); unfortunately, the MAX data used in this study do not contain National Physician Identification Numbers for the majority of the claims, making it impossible to link our analytical dataset with other datasets with physician specialty information.^33^

The delicate balance of risks and benefits of antidepressants (and other psychotropic medications) complicate the implications from our findings. Although there is a robust body of evidence demonstrating the effectiveness of psychotropic medication in the treatment of mood, psychotic, anxiety, developmental, and behavioral disorders,^34^ psychotropic medications can cause severe side effects, particularly SSRIs prescribed to young patients.^35^, ^36^ Therefore, the net clinical effect of reduced use of these medications (and a reduction in racial/ethnic differences in antidepressant use) in response to the box warning is not obvious, especially when baseline utilization levels vary across groups. That patients and their families do contribute significantly to decision making is notable and perhaps attributable to the fact that patients and families were very likely exposed to the significant coverage related to the box warning in news outlets and in the popular press.^21^

We offer evidence that providers contribute to larger shifts in prescribing antidepressants for Whites as compared to Black and Latino patients, in relative and absolute terms, as differences in the speed of risk diffusion varied by the racial/ethnic composition of the patient panel of providers. The reaction to the box warning was slower among providers with more than 2/3 minority patients compared with providers with panels of predominantly White patients. This result is consistent with prior studies showing that providers of racial/ethnic minority patients are less likely to be influenced by the research findings of the FDA and other organizations than providers of White patients.^37^ These findings also echo prior studies in the adult patient literature demonstrating that providers serving predominantly minority patients provide potentially lower quality of care than providers serving predominantly White patients.^11^, ^12^, ^13^ Additionally, the family and individual preferences of racial/ethnic minorities to prefer talk therapy over antidepressants,^17^ poorer communication between clinicians and their racial/ethnic minority patients, and statistical discrimination toward minority patients^38^, ^39^ may play important roles in the differential influence of providers.

In conclusion, this study identified that the reduction in racial/ethnic differences in antidepressant use after the box warning (caused by the decline among White youth and steady rates among Blacks and Latinos) was in large part explained by the fact that racial/ethnic minority youth largely seek care from a different subset of providers than White youth and that minority‐serving providers reacted differently to the box warning. As rates of psychotropic medication use in youth continue to climb in the United States,^40^ information about the long‐term risks of these medications has emerged eg,^41^, ^42^ and will continue to challenge decision making for providers, youth living with mental illness, and their caregivers. Going forward, policy makers and prescribers should pay close attention to how these risk warnings are disseminated and the differential practice patterns of providers of youth of color, and target resources and provider education to lower the risk of adverse health outcomes faced by these vulnerable patient populations.

## ENDNOTE

1 We choose to use *difference* as opposed to *disparity* when discussing racial/ethnic differences in antidepressant use in order to remain agnostic about the value of an increase or decrease in antidepressant use. While there is some debate over the risks and benefits of the box warning and changes in antidepressant use, we are concerned in this study with the influence of the warning on provider and patient behavior as opposed to assessing whether the warning was beneficial or not.

## References

[1] Gibbons R , Brown C , Hur K , et al. Early evidence on the effects of regulators’ suicidality warnings on SSRI prescriptions and suicide in children and adolescents. Am J Psychiatry. 2007;164(9):1356‐1363. 17728420 10.1176/appi.ajp.2007.07030454

[2] Nemeroff CB , Kalali A , Keller MB . Impact of publicity concerning pediatric suicidality data on physician practice patterns in the United States. Arch Gen Psychiatry. 2007;64:466‐472. 17404123 10.1001/archpsyc.64.4.466

[3] Libby A , Brent D , Morrato E , Orton H , Allen R , Valuck R . Decline in treatment of pediatric depression after FDA advisory on risk of suicidality with SSRIs. Am J Psychiatry. 2007;164(6):884‐891. 17541047 10.1176/ajp.2007.164.6.884

[4] Weiss AP . Measuring the impact of medical research: moving from outputs to outcomes. Am J Psychiatry. 2007;164:206‐214. 17267781 10.1176/ajp.2007.164.2.206

[5] Flowers CR , Fedewa SA , Chen AY , et al. Disparities in the early adoption of chemo‐immunotherapy for diffuse large B‐cell lymphoma in the United States. Cancer Epidemiol Biomarkers Prev. 2012;21(9):20. 22771484 10.1158/1055-9965.EPI-12-0466 PMC4155492

[6] Buchanan A , Cole T , Keohane RO . Justice in the diffusion of innovation. J Polit Philos. 2011;19:306‐332.

[7] DePetris AE , Cook BL . Differences in diffusion of FDA antidepressant risk warnings across racial‐ethnic groups. Psychiatr Serv. 2013;64(5):466‐471, 471 e461–e464. 23412363 10.1176/appi.ps.201200087 PMC3686566

[8] Carson NJ , Progovac AM , Wang Y , Cook BL . A decline in depression treatment following FDA antidepressant warnings largely explains racial/ethnic disparities in prescription fills. Depress Anxiety. 2017;34(12):1147‐1156. 28962069 10.1002/da.22681 PMC5895183

[9] Guagliardo MF , Ronzio CR , Cheung I , Chacko E , Joseph JG . Physician accessibility: an urban case study of pediatric providers. Health Place. 2004;10(3):273‐283. 15177201 10.1016/j.healthplace.2003.01.001

[10] Hayanga AJ , Waljee AK , Kaiser HE , Chang DC , Morris AM . Racial clustering and access to colorectal surgeons, gastroenterologists, and radiation oncologists by African Americans and Asian Americans in the United States a county‐level data analysis. Arch Surg. 2009;144(6):532‐535. 19528386 10.1001/archsurg.2009.68

[11] Bach PB , Pham HH , Schrag D , Tate RC , Hargraves JL . Primary care physicians who treat blacks and whites. N Engl J Med. 2004;351(6):575‐584. 15295050 10.1056/NEJMsa040609

[12] Barnato AE , Lucas FL , Staiger D , Wennberg DE , Chandra A . Hospital‐level racial disparities in acute myocardial infarction treatment and outcomes. Med Care. 2005;43(4):308. 15778634 10.1097/01.mlr.0000156848.62086.06 PMC2121607

[13] Hasnain‐Wynia R , Baker DW , Nerenz D , et al. Disparities in health care are driven by where minority patients seek care: examination of the hospital quality alliance measures. Arch Intern Med. 2007;167(12):1233‐1239. 17592095 10.1001/archinte.167.12.1233

[14] Basu A . Economics of individualization in comparative effectiveness research and a basis for a patient‐centered health care. J Health Econ. 2011;30(3):549‐559. 21601299 10.1016/j.jhealeco.2011.03.004 PMC3110511

[15] King JS , Eckman MH , Moulton BW . The potential of shared decision making to reduce health disparities. J Law Med Ethics. 2011;2011(30):6. 10.1111/j.1748-720X.2011.00561.x 21309892

[16] Anderson LE , Chen ML , Perrin JM , Van Cleave J . Outpatient visits and medication prescribing for US children with mental health conditions. Pediatrics. 2015;136:e1178‐e1185. 26459647 10.1542/peds.2015-0807 PMC4621795

[17] Cooper LA , Gonzales JJ , Gallo JJ , et al. The acceptability of treatment for depression among African‐American, Hispanic, and white primary care patients. Med Care. 2003;41:479‐489. 12665712 10.1097/01.MLR.0000053228.58042.E4

[18] Miranda J , Cooper L . Disparities in care for depression among primary care patients. J Gen Intern Med. 2004;19(2):120‐126. 15009791 10.1111/j.1525-1497.2004.30272.x PMC1492138

[19] Stockdale SE , Lagomasino IT , Siddique J , McGuire T , Miranda J . Racial and ethnic disparities in detection and treatment of depression and anxiety among psychiatric and primary health care visits, 1995‐2005. Med Care. 2008;46:668‐677. 18580385 10.1097/MLR.0b013e3181789496 PMC3956700

[20] Hesse BW , Nelson DE , Kreps GL , et al. Trust and sources of health information: the impact of the Internet and its implications for health care providers: findings from the first Health Information National Trends Survey. Arch Intern Med. 2005;165(22):2618. 16344419 10.1001/archinte.165.22.2618

[21] Barry CL , Busch SH . News coverage of FDA warnings on pediatric antidepressant use and suicidality. Pediatrics. 2010;125(1):88‐95. 19969614 10.1542/peds.2009-0792 PMC2805031

[22] van‘t Riet J , Ruiter RAC . Defensive reactions to health‐promoting information: an overview and implications for future research. Health Psychol Rev. 2013;7(supp1):34.

[23] Cheung A , Sacks D , Dewa CS , Pong J , Levitt A . Pediatric prescribing practices and the FDA black‐box warning on antidepressants. J Dev Behav Pediatr. 2008;29:3. 10.1097/DBP.0b013e31817bd7c9 18550990

[24] Lacy BW , Baron DA , Goin MK , Gross LS , Slaby AE , Sumner CR . Doing nothing is doing something: primary care antidepressant treatment in the era of FDA boxed warnings. J Am Osteopath Assoc. 2013;113(2):3. 23412671

[25] Parkinson K , Price J , Simon KI , Tennyson S . The influence of FDA advisory information and black box warnings on individual use of prescription antidepressants. Rev Econ Household. 2014;12:771‐770.

[26] Morris NS , Grant S , Repp A , MacLean C , Littenberg B . Prevalence of limited health literacy and compensatory strategies used by hospitalized patients. Nurs Res. 2011;60(5):6. 10.1097/NNR.0b013e31822c68a6 PMC3212986 21878798

[27] Dusetzina SB , Busch AB , Conti RM , Donohue JM , Alexander GC , Huskamp HA . Changes in antipsychotic use among patients with severe mental illness after a Food and Drug Administration advisory. Pharmacoepidemiol Drug Saf. 2012;21:10. 10.1002/pds.3272 PMC3431449 22553074

[28] Raudenbush SW , Bryk AS . Hierarchical Linear Models: Applications and Data Analysis Methods, 2nd edn London: Sage Publications; 1992.

[29] Hasnain‐Wynia R , Baker DW , Nerenz D , et al. Disparities in health care are driven by where minority patients seek care: examination of the hospital quality alliance measures. Arch Intern Med. 2007;167(12):1233. 17592095 10.1001/archinte.167.12.1233

[30] Bekelis K , Skinner J , Gottlieb D , Goodney P . De‐adoption and exnovation in the use of carotid revascularization: retrospective cohort study. BMJ. 2017;359:j4695. 29074624 10.1136/bmj.j4695 PMC5656975

[31] Fennell ML . Racial disparities in care: looking beyond the clinical encounter. Health Serv Res. 2005;40(6 Pt 1):1713. 16336544 10.1111/j.1475-6773.2005.00489.x PMC1361239

[32] Smith DB . Health Care Divided: Race and Healing a Nation. Ann Arbor, MI: The University of Michigan Press; 1999.

[33] Centers for Medicare & Medicaid Services . MAX provider characteristics. 2018 https://www.cms.gov/Research-Statistics-Data-and-Systems/Computer-Data-and-Systems/MedicaidDataSourcesGenInfo/MAXPC.html. Accessed July 25, 2018.

[34] Martin A , Scahill L , Kratochvil C . Pediatric Psychopharmacology. New York, NY: Oxford University Press; 2010.

[35] Merikangas KR , He J‐P , Rapoport J , Vitiello B , Olfson M . Medication use in US youth with mental disorders. JAMA Pediatrics. 2013;167(2):141‐148. 23403911 10.1001/jamapediatrics.2013.431

[36] Bridge JA , Salary CB , Birmaher B , Asare AG , Brent DA . The risks and benefits of antidepressant treatment for youth depression. Ann Med. 2005;37(6):404‐412. 16203613 10.1080/07853890500284937

[37] Fennell ML , Adams C . U.S. health‐care organizations: complexity, turbulence, and multilevel change. Ann Rev Sociol. 2011;37:20.

[38] Balsa A , McGuire T . Statistical discrimination in health care. J Health Econ. 2001;20(6):881‐907. 11758051 10.1016/s0167-6296(01)00101-1

[39] Balsa A , McGuire T , Meredith L . Testing for statistical discrimination in health care. Health Serv Res. 2005;14(1):227‐252. 10.1111/j.1475-6773.2005.00351.x PMC1361135 15663711

[40] Olfson M , Druss BG , Marcus SC . Trends in mental health care among children and adolescents. N Engl J Med. 2015;372(21):2029‐2038. 25992747 10.1056/NEJMsa1413512

[41] Correll CU , Joffe BI , Rosen LM , Sullivan TB , Joffe RT . Cardiovascular and cerebrovascular risk factors and events associated with second‐generation antipsychotic compared to antidepressant use in a non‐elderly adult sample: results from a claims‐based inception cohort study. World Psychiatry. 2015;14(1):56‐63. 25655159 10.1002/wps.20187 PMC4329898

[42] Correll CU , Manu P , Olshanskiy V , Napolitano B , Kane JM , Malhotra AK . Cardiometabolic risk of second‐generation antipsychotic medications during first‐time use in children and adolescents. JAMA. 2009;302(16):1765‐1773. 19861668 10.1001/jama.2009.1549 PMC3055794

